# The influence of interradicular anatomy on the predictability of periodontal regenerative therapy of furcation defects: a retrospective, multicenter clinical study

**DOI:** 10.1007/s00784-023-04995-3

**Published:** 2023-04-13

**Authors:** Enrico Limiroli, Andrea Calò, Pierpaolo Cortellini, Peter Eickholz, Akihiko Katayama, Jad Majzoub, Jennifer Wong, Pamela McClain, Ivan Cortinovis, Giulio Rasperini

**Affiliations:** 1 Department of Biomedical, Surgical and Dental Sciences, University of Milan, Milan, Italy; 2 Foundation IRCCS Ca’ Granda Polyclinic, Via Della Commenda 10, 20122 Milan, Italy; 3 Private Practice, Florence, Italy; 4 Department of Periodontology, Center for Dentistry and Oral Medicine (Carolinum), Johann Wolfgang Goethe-University Frankfurt Am Main, 60596 Frankfurt Am Main, Germany; 5 Private Practice, Tokyo, Japan; 6 Department of Periodontics and Oral Medicine, University of Michigan, Ann Arbor, MI USA; 7 Department of Oral Medicine, Infection, and Immunity, Division of Periodontology at the Harvard School of Dental Medicine, Boston, MA USA; 8 Private Practice, Aurora, CO USA; 9 Laboratory G.A. Maccacaro, Department of Clinical Sciences and Community Health, University of Milan, Milan, Italy

**Keywords:** Furcation defect furcation treatment, Root divergence, Furcation anatomy, Regenerative therapy, Furcation regeneration

## Abstract

**Background:**

The relationship between the anatomy of the interradicular space and success in regenerative therapy of furcation defects is discussed in this paper. The goal of this retrospective, multicenter clinical study is to clinically evaluate the relationship between the interradicular conformation and regenerative therapy success with the use of a novel measurement method.

**Methods:**

One hundred thirty-eight radiographs of mandibular molars with furcation defects that had been treated with regenerative therapy were collected from six clinical centers. Data on the type of therapy and clinical parameters before and after treatment (follow-up of at least 12 months) were collected. The radiographs (before surgery and at least 12 months postoperatively) were measured with a visual evaluation method by a blind operator using graphics software.

**Results:**

Success, defined as a reduction in horizontal and vertical furcation involvement, decrease in probing depths, and increase in clinical attachment level, was statistically assessed on 138 regenerated molars sites and were related to clinical variables such as age, sex, center, and treatment. No correlation was found between success in regenerative therapy and the conformation of the interradicular space, measured with a visual ratio method and a standard linear measurement.

At the univariate analysis, the parameters that had a correlation with success were center, extent of furcation involvement, treatment, and sex. The use of enamel matrix derivative (EMD) seemed to be the most favorable therapy, with increase in CAL gain and reduction of vertical or horizontal furcation involvement.

**Conclusions:**

The regenerative outcome was not significantly influenced by the anatomy of furcation. The center, the degree of furcation involvement, sex, and treatment (EMD) were significantly associated with higher success of periodontal regeneration.

## Introduction

Regenerative therapy of furcation defects is one of the biggest challenges of periodontal surgery. Different regenerative strategies have been proposed to improve the prognoses of teeth with furcation involvement (FI), including guided tissue regeneration [1], bone grafting [2], and using enamel matrix derivative (EMD) [3]. Although achieving complete bone fill of the furcation defect is difficult when treating class II furcations, studies have shown that partial bony regeneration of the furcation defect, with the consequent conversion from a Class II to a Class I defect, might improve the prognosis of the tooth over time [4, 5]. From a biological viewpoint, the regenerative potential within the furcation area is limited. The clinical outcomes of periodontal surgical therapy can be negatively affected by several anatomical factors, such as the extent of the bone loss in the furcation, both in the horizontal [6] and vertical [7] dimensions [8]; the interproximal bone level; the width of keratinized gingiva; the gingival thickness [9, 10]; and the anatomy of the roots [11–14]. Some studies found that the divergence of the furcation and the length of the root trunk were correlated to the success of periodontal regenerative therapy at furcation defects [11–14], while other authors did not confirm this finding [15]. There is relatively little in the literature regarding the average root divergence and the width of the inter-root septum. However, it is reasonable to assume that this anatomy of the root may play a key role in the regeneration potential of the furcation defect. Several articles discuss measuring the parameters that characterize the anatomy of the roots, such as the furcation divergence, root trunk length, radicular roof width, and interradicular width [12–17]. Bowers and coworkers proposed a definition of root divergence of mandibular molars as the horizontal distance between the two roots at the level of the crestal bone [12]. Another method of evaluating the root divergence was later proposed by Caesarin et al. [18] as the distance between the roots at 2 mm apical to the fornix of the furcation. One limitation of these methods is the need for these measurements to be performed intrasurgically, i.e., following flap elevation and debridement of the furcation defect. Another limitation is the fact that the measurement of root divergence is carried out at a single reference point, overlooking any root divergence coronal or apical to this reference point. For example, in the case scenario of a tooth with root cones that converge more apically to the reference points suggested by Bowers et al. [12] and Caesarin et al. [18], this convergence would not be considered when using their methods of describing root divergence. A further limitation is that the measurement of root divergence is purely linear, ignoring the three-dimensional space created by the furcation roof and the divergence of the roots, where the horizontal distance varies depending on the reference point.

We recently proposed a new method to measure the interradicular anatomy [24] taking into account the radicular roof width and interradicular width. The goal of this article is to evaluate the impact of the interradicular anatomy of mandibular molars on the clinical outcomes of periodontal regenerative therapy of furcation defects.

## Materials and methods

### Study design

This is a multicenter, retrospective clinical study with the main goal of investigating a possible relationship between interradicular conformation and the clinical outcomes of regenerative therapy. The data were collected from six clinical centers. A novel evaluation method [24] was used to assess and measure the interradicular space of mandibular molars with FI that had been previously treated with periodontal regenerative therapies. The interradicular space was measured using periapical radiographs taken at baseline and at follow-up at least 12 months later.

### Inclusion criteria

Radiographs were included in the present study only if they met the following inclusion criteria:

i. Radiographs taken with a paralleling technique
ii. Good image definition (root margins clearly distinguishable)
iii. Complete view of the interradicular space and root apices
iv. Infrabony defect of at least 3 mm
v. Absence of endodontic lesions
vi. Interproximal bone level coronal or at the same level as the roof of the furcation

### Data collection

A total of 138 periapical radiographs of mandibular molars that met the inclusion criteria and that had been treated with regenerative therapy were collected from six clinical centers. The following de-identified data were retrieved from each center: (i) regenerative technique performed; (ii) patient demographic characteristics, including age and sex; (iii) initial and final vertical probing depth (PD) at the midfacial aspect; (iv) midfacial gingival recession depth (REC); (v) midfacial clinical attachment level (CAL), obtained adding REC to PD; and (vi) horizontal and vertical furcation class at the last follow-up. The success of the therapy was defined as the presence of clinical attachment level gain and concomitant improvement of the FI (horizontal and/or vertical reduction of the furcation defect) compared to baseline.

A calibrated operator who was unaware of the center, the technique performed or the final clinical outcome, carried out measurements on X-rays of interradicular conformation using the method described by Limiroli et al. [24] and the degree of vertical FI [19]. Being a retrospective study, the single centers were not calibrated, and clinical data and X-rays were sent to a collecting center, where a calibrated operator interpreted the data and took the measurement on the X-rays. Briefly, this method evaluated the interradicular anatomy with graphics software (in this study, Paint®, Windows10®, Microsoft, USA was used) utilizing ratios derived from a bi-dimensional evaluation of the interradicular space:

Two ratios were calculated:

- Roof furcation width (RFW): the ratio between the diameter of the osculating circle (it is the best circle that approximates a curve; in this case, it is a circle overlapping as much as possible the furcation roof curvature, overlapping the roof of the furcation and the length of the line from the roof of the furcation to the midpoint of an imaginary line passing through the tips of the roots) and the line from the roof of the furcation to an imaginary line passing through the tips of the roots (vertical line).
- Interradicular furcation width (IFW): the ratio between a horizontal line drawn at the maximum width of the interradicular septum and the vertical line.

**Fig. 1:**
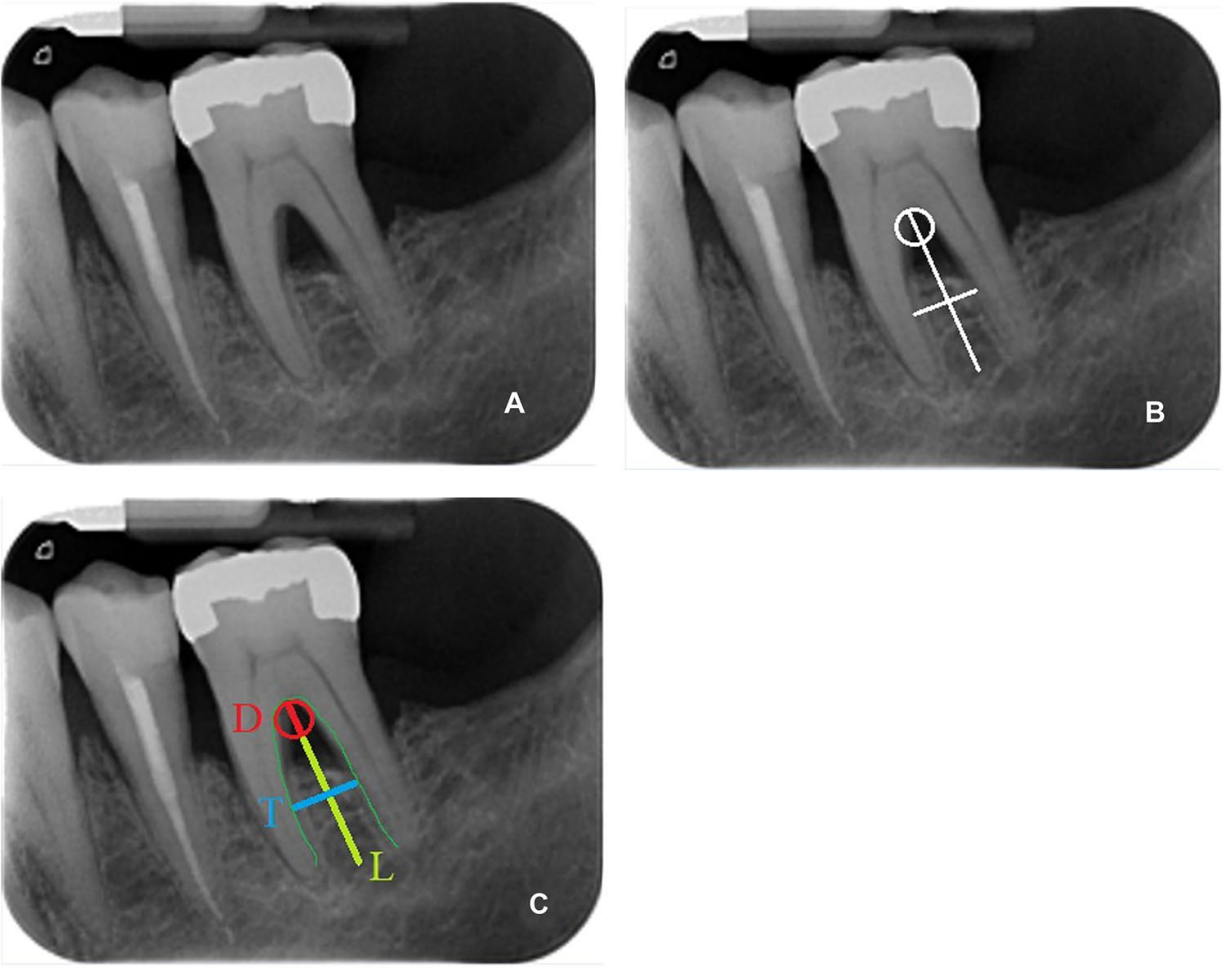
Example of root divergence assessment. **A** Peri-apical X-ray. **B** Application of the “visual” evaluation method for assessing root divergence. **C**) The osculating circle (D), the transversal width (T), and the longitudinal length (L) are depicted in the X-ray. RFW is obtained from the ratio of D and L, while IFW is obtained from the ratio of T and L. Picture from Limiroli et al. [24]

The two ratios represent the radicular roof width and the interradicular width (Fig. 1).

In addition to the newly proposed method, the interradicular conformation was also assessed using the method described by Bowers [12]: in this method, the divergence was identified with the linear distance of the roots at the height of the defect crestal bone. As a control for the RFW/IFW measurement technique, this standard linear measure (MR) of root divergence (Bowers et al. [12]) was applied directly to the radiographic images.

### Statistical analysis

The collected data were described using measures of central tendency and dispersion. Quintile values were calculated for interradicular conformation measurements (RFW, IFW, MR). The relationship between these variables was also investigated using the linear correlation coefficient. The relationship between the interradicular conformation measurements and treatment outcomes was evaluated with these as control variables: center, sex, age, and type of treatment. A multivariable logistic model was used to study this relationship in the descriptive tables. The association between the variables was measured with the chi-squared test. The statistical significance threshold was set at *p* = 0.05.

## Results

Six centers provided 138 radiographs of 138 mandibular molars with FI treated with periodontal regeneration (Table 1). Seventy-six sites were treated with GTR, 34 with EMD, and 10 with fibroblast growth factor 2 (FGF-2).

**Table 1:** Study population and baseline characteristics for each center

	Subjects (*N*)	Females (*N*)/ (%)	Age (mean ± SD) (years)	CAL (mean ± SD) (mm)	PD (mean ± SD) (mm)
Center 1	23	11/ 47.83	53.83 ± 8.59	10.43 ± 3.33	8.52 ± 2.76
Center 2	20	10/ 50	43.95 ± 10.65	5.30 ± 2.07	4.55 ± 1.91
Center 3	15	14 / 93.33	47.80 ± 11.91	7.30 ± 1.54	5.67 ± 1.05
Center 4	18	11/ 61.11	50.33 ± 10.62	7.78 ± 1.40	6.56 ± 1.42
Center 5	15	10/ 66.67	51.93 ± 3.81	7.20 ± 2.04	5.60 ± 1.80
Center 6	47	N/A	N/A	5.49 ± 0.78	5.04 ± 0.83
Overall	138	56/ 61.54	49.66 ± 10.04	6.97 ± 2.60	5.88 ± 2.10

*CAL* clinical attachment level, *N/A* not available, *PD* pocket depth, *SD* standard deviation

The distribution of cases exhibiting CAL gain (Rec plus PD) and those also obtaining an improvement of the furcation involvement (“successful cases”) after treatment are depicted in Table 2. The centers 2 and 6 had a substantial lower incidence of successful cases (35% and 57.45%, respectively) compared to others (successful cases ranging from 80 to 94.44%). While the reduction of the FI was not associated to the center, the statistical analysis showed a significant correlation between the center and the treatment outcomes, in terms of successful cases and CAL gain (Table 2).

**Table 2:** Incidence of cases that resulted in success and CAL gain

	Subjects (*N*)	Successful cases* (*N*)/(%)	Cases exhibiting CAL gain (mean ± SD) (mm)
Center 1	23	21/ 91.30	23/ 100
Center 2	20	7/ 35.00	12/ 60.00
Center 3	15	14 / 93.33	14/ 93.33
Center 4	18	17/ 94.44	18/ 100.00
Center 5	15	12/ 80.00	13/ 86.67
Center 6	47	27/ 57.45	36/ 76.60
Overall	138	98/ 71.01	116/ 84.06

^*^Cases were defined successful if they showed at the last follow-up clinical attachment level gain and concomitant improvement of the furcation involvement (horizontal and/or vertical reduction of the furcation defect) compared to baseline

**Fig. 2:**
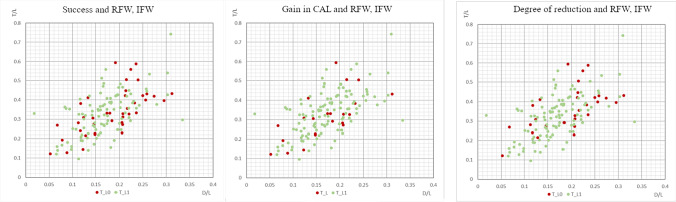
Graphs illustrating the distribution of RFW and IFW in relation to the success of the treatment, CAL gain, and degree of reduction of the furcation involvement. The green dots display the successful cases (CAL gain and reduction of the furcation involvement), while the red dots represent the unsuccessful cases

The interradicular space conformation (RFW and IFW) was calculated on the 138 radiographs. The values of both ratios in study population overlapped the conformation range and distribution of a standard population. Table 3 and Fig. 2 describe the association between the interradicular space conformation, and the cases defined successful, cases exhibiting CAL gain, and cases resulting in a reduction of the degree of the FI. Once the confidence mean and interval was calculated, it was noted that the ranges measurements were overlapping, leading to the conclusion that there is no statistically significant difference between the values of RFW and IFW distributed by the above parameters (Fig. 2).

**Table 3:** Influence of the interradicular conformation measures on the clinical and radiographic outcomes

		Successful cases		CAL gain		Furcation degree reduction	
		No (*N* = 39)	Yes (*N* = 86)	No (*N* = 21)	Yes (*N* = 104)	No (*N* = 29)	Yes (*N* = 96)
MR	*Mean*	0.982	1.089	0.9952	1.0683	0.9827	1.0781
	*CI 95%*	0.819–1.145	0.967–1.212	0.765–1.225	0.959–1.773	0.803–1.162	0.962–0.181
RFW	*Mean*	0.187	0.170	0.171	0.176	0.193	0.169
	*CI 95%*	0.166–0.206	0.158–0.181	0.142–0.199	0.165–0.187	0.169–0.218	0.159–0.180
IFW	*Mean*	0.346	0.321	0.323	0.330	0.366	0.317
	*CI 95%*	0.309–0.383	0.298–0.343	0.269–0.377	0.309–0.350	0.324–0.409	0.296–0.338

*CAL* clinical attachment level, *CI* confidence interval, *IFW* interradicular furcation width, *MR* standard linear measure of root divergence, *RFW* roof furcation width

^*^Cases were defined successful if they showed at the last follow-up clinical attachment level gain and concomitant improvement of the furcation involvement (horizontal and/or vertical reduction of the furcation defect) compared to baseline)

Parameters were then considered in a combined manner, and the sample of subjects resulted was divided into four groups. The first group of 11 subjects was the unsuccessful one, presenting a lack of CAL gain and no degree of reduction of furcation involvement. The second group of 11 subjects presented only degree of reduction of furcation involvement. The third group of 18 subjects showed only CAL gain. The fourth group of 98 subjects demonstrated all 3 positive variables success, degree of reduction, and gain in CAL. Distribution of RFW and IFW related to the groups was then assessed. The overlapping confidence intervals showed no significant association. The same groups described above were then compared with the variables horizontal FI and vertical. The result confirmed the previous association between success and reduction of furcation involvement variables. Moreover, crossing both the variables horizontal and vertical FI with success, it was concluded that 97% of subjects presenting success in the regenerative procedure also obtained an improvement in both vertical and horizontal furcation.

Degree III horizontal furcation defects, considered to be subject to more difficulty in obtaining regeneration, were then removed, to evaluate a possible negative influence. The relationship between variables horizontal/vertical FI and RFW/IFW was considered: unsuccess resulted in a lower number confirming the III degree furcations as the more uncertain to be treated with regeneration, but even in this scenario, the confidence interval values were overlapping for both groups, not recording a statistically significant difference in the influence of RFW and IFW even without degree III horizontal furcation defects. The association between RFW and IFW relating to the variables Hf and Vf was related to success or unsuccess. The overlapping values of the confidence intervals indicated no statistically significant association.

At this point, as a control to the RFW/IFW measurement technique, a standard linear measure (MR) of root divergence (Bowers et al. [17]) was applied directly on radiographic images. Relationship between this standard method and success was analyzed. Statistically, overlapping in the confidence intervals results was found, showing no association between MR and success.

Finally, a regression model was then created considering the only variable type of treatment. Using a multivariable logistic regression model as response variable “yes” was considered the success variable, and as independent variables gender, age, treatment type and, for each one of the results, one of the 3 interradicular conformation variables. Statistically significant associations were not found. The exception was the sex variable in the model that considers the variable RFW. In all 3 logistic regression models considered, the variables RFW, IFW, and MR were express in quintiles.

To establish how much the different factors are involved in the regenerative outcomes, a factor influence ranking was used. The associations between covariates and outcomes were investigated with a univariate analysis.

The variables associated with the success full cases were, in order of strength of association: center effect, horizontal FI, vertical FI, treatment, and sex (with more favorable outcomes observed in females). Therefore, the center was the variable strongly associated with the success of the regenerative outcomes of mandibular molars with FI. Age, RFW, and IFW were not found instead be associated with the outcome success.

## Discussion

It has been suggested that the interradicular anatomy is associated with the outcomes of periodontal regeneration [11–18]. Several articles have described methods for assessing different parameters defining the interradicular anatomy, such as root divergence, root trunk length, radicular roof width, and interradicular width [12–17]. The aim of the present study was to evaluate the relationship between the interradicular space anatomy and the clinical outcomes of regenerative therapy. We utilized a recently proposed method that advocates the use of two ratios (RFW and IFW) for characterizing the conformation of the furcation and the entire length of the root anatomy [24].

Our finding was that the outcome of periodontal regeneration of furcation defects was not affected by the interradicular anatomy. The lack of association was observed when the interradicular anatomy was assessed using the conventional method and when the assessment was performed with the use of the two ratios. On the other hand, the analysis showed that the outcome of regenerative therapy of furcation defects was affected by the technique, center effect, type of horizontal/vertical FI, and sex. In addition, regenerative therapies utilizing EMD achieved the highest CAL gain, FI improvement, and, overall, the highest probability of treatment success.

The effectiveness of EMD has been evaluated by several clinical studies over the last 20 years [19, 20]. EMD is referred to as one of the most widely used therapies for the treatment of infrabony defects, with documented success in the literature. Cortellini and Tonetti in 2020 [21] examined the action of EMD after performing a papilla conservation flap (PPF) in class II and class III furcation defects. They found some improvement in CAL, PPD, and the horizontal and vertical component in both Class II and Class III furcations.

The grade of the horizontal and/or vertical component of the furcation defect affects the prognosis of molars with FI and is a key parameter when evaluating the outcomes of periodontal regeneration [22, 23]. Tonetti in 2017 [7] evaluated bone loss by using periapical radiographs taken with the parallel technique and conducted clinical examination of PD and CAL. The vertical classification was based on the area with the highest radiographic bone loss, creating three subclasses A, B, and C, with prognosis worsening from A to C. It was noted that molars with periodontal furcation involvement were lost or extracted earlier and more frequently if the loss of support reached the apical portion of one of the roots (subclass C). Through survival curves, it was shown that the survival of teeth with vertical furcation involvement decreased from subclass A to C. The author reported that the 10-year survival rate was 91% for teeth with subclass A, 67% for subclass B, and 23% for subclass C furcation defects. It was interesting to observe that in the present study, 97% of the subjects who obtained success also presented an improvement to the horizontal and vertical involvement simultaneously.

The IFW and RFW values calculated from the 138 radiographs were consistent with those of the population sample examined in a recent study from our group [24]. Given the previously available scientific literature showing that the furcation and interradicular anatomy plays a decisive role on the outcome of regenerative therapy [17], our hypothesis was that the RFW and IFW, which together express the conformation of the interradicular space, were significantly associated with reduction of FI since, as supposed in literature, a wider furcation could improve regeneration potential of the defect and, overall, with the success of the surgical treatment. However, the statistical analysis failed to support this hypothesis. The reasons for this finding are open to speculation. It is possible that expressing the divergence as a ratio may not describe the complexity of the interradicular anatomy in certain case scenarios. Interestingly, when the morphology of the furcation/roots was evaluated as a single linear measurement expressing root divergence at the level of the crestal bone, as suggested in classical studies [12–18], the relationship between the outcomes/success of periodontal regeneration and the interradicular anatomy did not change, further supporting the lack of influence of this parameter on the surgical results regardless of the methodology utilized.

Finally, the statistical analysis was able to highlight, using a factor influence ranking, that the strongest effect on the treatment outcomes was obtained by the center effect, followed by the type of FI, treatment, and sex. The possible influence of furcation morphology on the effectiveness of the treatment was analyzed by Meyle et al. [25], with results in line with the present study. The authors found that furcation morphology at the time of surgery was not associated with clinical outcome, irrespective of the treatment. This means that furcation conformation does not represent a limit in furcation regeneration success.

Within the limitations of the present study, it should be mentioned that some of the radiographs may not have been performed in the ideal manner with the correct inclination between the X-ray beam and the film, although the paralleling technique could reduce the amount of distortion in the images. In addition, both the small sample size and the heterogenous nature of the clinical centers (university versus private practice) could have affected our findings. Future prospective clinical studies evaluating the influence on the interradicular anatomy assessed with conventional and novel methods are encouraged.

## Conclusions

Within the limitations of the present study, it can be concluded that interradicular anatomy does not seem to affect the outcomes of periodontal regenerative therapy. EMD was found to be the most effective treatment approach in terms of CAL gain and reduction of the horizontal and/or vertical FI. Center effect, horizontal/vertical FI at baseline, and sex were also significantly associated with the surgical outcomes.

## References

[1] Jepsen S, Eberhard J, Herrera D, Needleman I (2002). A systematic review of guided tissue regeneration for periodontal furcation defects What is the effect of guided tissue regeneration compared with surgical debridement in the treatment of furcation defects?. J Clin Periodontol.

[2] Jepsen S, Gennai S, Hirschfeld J, Kalemaj Z, Buti J, Graziani F (2020). Regenerative surgical treatment of furcation defects: a systematic review and Bayesian network meta-analysis of randomized clinical trials. J Clin Periodontol.

[3] Koop R, Merheb J, Quirynen M (2012). Periodontal regeneration with enamel matrix derivative in reconstructive periodontal therapy: a systematic review. J Periodontol.

[4] Trombelli L, Farina R (2008). Clinical outcomes with bioactive agents alone or in combination with grafting or guided tissue regeneration. J Clin Periodontol.

[5] McGuire MK, Nunn ME (1996). Prognosis versus actual outcome .III. The effectiveness of clinical parameters in accurately predicting tooth survival. J Periodontol.

[6] Hamp SE, Nyman S, Lindhe J (1975). Periodontal treatment of multirooted teeth. Results after 5 years. J Clin Periodontol.

[7] Tonetti M, Christiansen A, Cortellini P (2017). Vertical subclassification predicts survival of molars with class II furcation involvement during supportive periodontal care. J Clin Periodontol.

[8] Nibali L, Sun C, Akcalı A, Yeh Y-C, Tu Y-K, Donos N (2018) The effect of horizontal and vertical furcation involvement on molar survival: a retrospective study. J Clin Periodontol 45:373–381 https://doi-org.pros1.lib.unimi.it/10.1111/jcpe.12850 10.1111/jcpe.12850 29219193

[9] Pilloni A, Rojas MA (2018) Furcation involvement classification: a comprehensive review and a new system proposal. Dent J (Basel) 6 10.3390/dj6030034 PMC6162379 30041399

[10] Anderegg CR, Metzler DG, Nicoll BK (1995). Gingiva thickness in guided tissue regeneration and associated recession at facial furcation defects. J Periodontol.

[11] Cortellini P, Cortellini S, Tonetti M (2019). Papilla preservation flaps for periodontal regeneration of molars severely compromised by combined furcation and intrabony defects: retrospective analysis of a registry-based cohort. J Periodontol.

[12] Bowers G, Schallhorn R, McClain P, Morrison G, Morgan R, Reynolds M (2003). Factors influencing the outcome of regenerative therapy in mandibular Class II furcations: Part I. J Periodontol.

[13] Eickholz P, Hausmann E (1997). Evidence for healing of class II and III furcations after GTR therapy: digital subtraction and clinical measurements. J Periodontol.

[14] Horwitz J, Machtei E, Reitmeir P, Holle R, Kim T, Eickholz P (2004). Radiographic parameters as prognostic indicators for healing of class II furcation defects. J Clin Periodontol.

[15] Meyle J, Gonzales JR, Bödeker RH, Hoffmann T, Richter S, Heinz B, Arjomand M, Reich E, Sculean A, Jepsen K, Jepsen S (2004). randomized clinical trial comparing enamel matrix derivative and membrane treatment of buccal class II furcation involvement in mandibular molars Part II: secondary outcomes. J Periodontol.

[16] Aichelmann-Reidy ME, Avila-Ortiz G, Klokkevold PR, Murphy KG, Rosen PS, Schallhorn RG (2015). Periodontal regeneration - furcation defects: practical applications from the AAP regeneration workshop. Clin Adv Periodontics.

[17] Rasperini G, Majzoub J, Tavelli L, Limiroli E, Katayama A, Barootchi S, Hill R, Wang HL (2020). Management of furcation-involved molars: recommendation for treatment and regeneration. Int J Periodontics Restorative Dent.

[18] Casarin RC, Ribeiro Edel P, Ribeiro FV, Nociti FH, Sallum AW, Sallum EA, Casati MZ (2009). Influence of anatomic features on the effectiveness of enamel matrix derivative proteins in the treatment of proximal Class II furcation involvements. Quintessence Int.

[19] Stavropoulos A, Bertl K, Spineli LM, Sculean A, Cortellini P, Tonetti M (2021). Medium- and long-term clinical benefits of periodontal regenerative/reconstructive procedures in intrabony defects: systematic review and network meta-analysis of randomized controlled clinical studies. J Clin Periodontol.

[20] Miron RJ, Sculean A, Cochran DL, Froum S, Zucchelli G, Nemcovsky C, Donos N, Lyngstadaas SP, Deschner J, Dard M, Stavropoulos A, Zhang Y, Trombelli L, Kasaj A, Shirakata Y, Cortellini P, Tonetti M, Rasperini G, Jepsen S, Bosshardt DD (2016). Twenty years of enamel matrix derivative: the past, the present and the future. J Clin Periodontol.

[21] Cortellini P, Cortellini S, Tonetti MS (2020). Papilla preservation flaps for periodontal regeneration of molars severely compromised by combined furcation and intrabony defects: retrospective analysis of a registry-based cohort. J Periodontol.

[22] Karring T, Nyman S, Gottlow J, Laurell L (1993). Development of the biological concept of guided tissue regeneration - animal and human studies. Periodontology.

[23] Eickholz P, Runschke M, Dannewitz B, Nickles K, Petsos H, Kronsteiner D, Pretzl B (2001) Long-term prognosis of teeth with class III furcation involvement. J Clin Periodontol 48(12) 1528–1536 https://doi-org.pros1.lib.unimi.it/10.1111/jcpe.13551 10.1111/jcpe.13551 34545596

[24] Limiroli E, Calò A, Limiroli A, Rasperini G (2022) Radiographic ratios for classifying furcation anatomy. Proposal of a new evaluation method and an intra-rater and inter-rater operator reliability study Accepted for publication, Clin Oral Investig 10.1007/s00784-022-04774-6 PMC10102072 36781478

[25] Meyle J, Gonzales JR, Bödeker RH, Hoffmann T, Richter S, Heinz B, Arjomand M, Reich E, Sculean A, Jepsen K, Jepsen S (2004). A randomized clinical trial comparing enamel matrix derivative and membrane treatment of buccal class II furcation involvement in mandibular molars Part II: secondary outcomes. J Periodontol.

